# The enzymes of microbial nicotine metabolism

**DOI:** 10.3762/bjoc.14.204

**Published:** 2018-08-31

**Authors:** Paul F Fitzpatrick

**Affiliations:** 1 Department of Biochemistry and Structural Biology, University of Texas Health Science Center, San Antonio, TX, 78229, USA

**Keywords:** biodegradation, enzyme mechanism, flavoprotein, metabolic pathway, nicotine

## Abstract

Because of nicotine’s toxicity and the high levels found in tobacco and in the waste from tobacco processing, there is a great deal of interest in identifying bacteria capable of degrading it. A number of microbial pathways have been identified for nicotine degradation. The first and best-understood is the pyridine pathway, best characterized for *Arthrobacter nicotinovorans*, in which the first reaction is hydroxylation of the pyridine ring. The pyrrolidine pathway, which begins with oxidation of a carbon–nitrogen bond in the pyrrolidine ring, was subsequently characterized in a number of pseudomonads. Most recently, a hybrid pathway has been described, which incorporates the early steps in the pyridine pathway and ends with steps in the pyrrolidine pathway. This review summarizes the present status of our understanding of these pathways, focusing on what is known about the individual enzymes involved.

## Introduction

The toxic alkaloid (*S*)-nicotine (L-nicotine) is found at high levels in tobacco leaves and the waste from tobacco processing. The resulting interest in developing environmentally friendly methods of degrading nicotine has driven studies of microbial pathways for metabolizing the compound, with the possible additional benefit of using the enzymes involved to synthesize specialty chemicals [1–2]. To date, the best-characterized bacterial pathways are those of *Arthrobacter nicotinovorans* and several pseudomonads. These are, respectively, known as the pyridine and pyrrolidine pathways due to the initial reactions in each. More recently, additional pathways have been described that combine steps from the pyridine and pyrrolidine pathways. To a large extent the descriptions of this metabolism have focused on the genes involved. An exception to this is the review by Brandsch [3], which describes the *Arthrobacter* pathway at a biochemical level. In the more than a decade since that review was published, a great deal has been learned about other pathways for nicotine metabolism and the enzymes involved. The goal of the present report is to summarize our present understanding of the different pathways by which microbes metabolize nicotine, focusing on the enzymes.

## Review

### The pyridine pathway

**Scheme 1:**
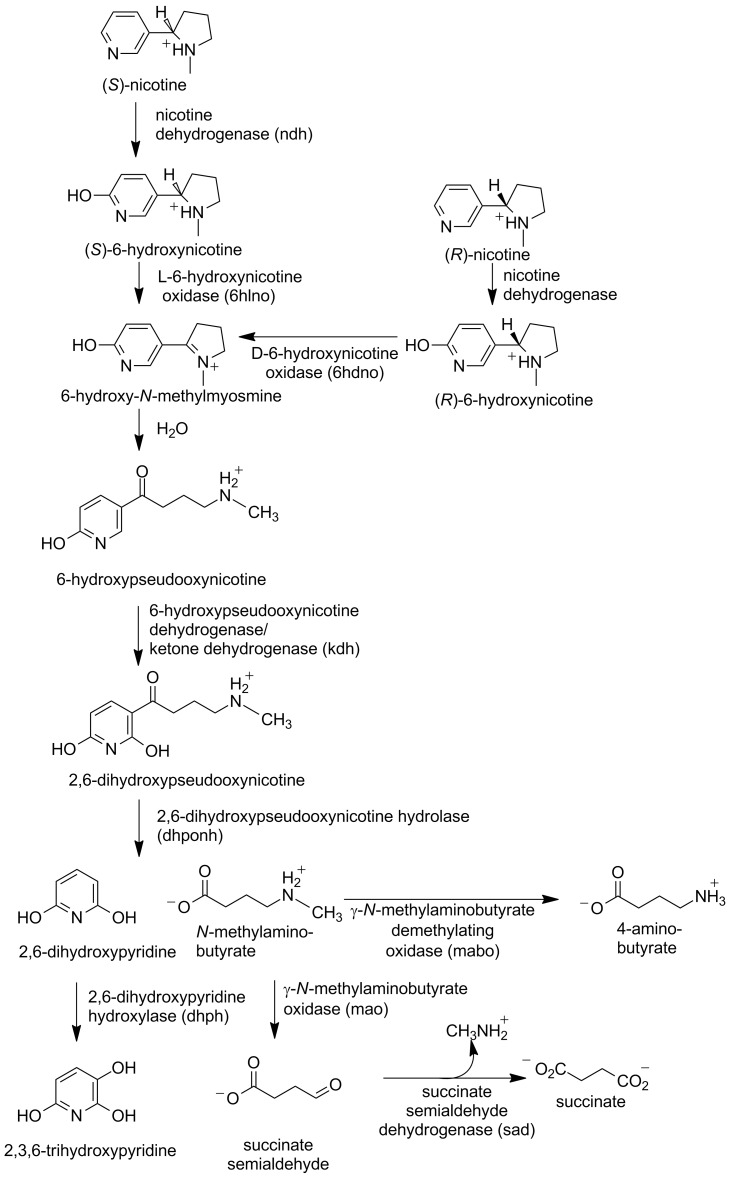
Nicotine catabolism in *A. nicotinovorans*. The respective gene names are given in parentheses.

**Scheme 2:**
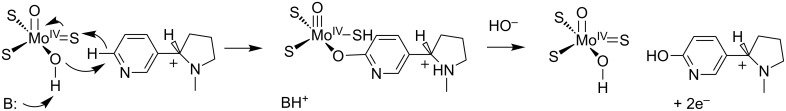
Hydroxylation of nicotine by the molybdopterin cofactor of nicotine dehydrogenase.

In *A. nicotinovorans* the enzymes involved in nicotine metabolism are found on the plasmid pAO1 [4], and the sequencing of this plasmid was a major step in elucidating the pathway [5]. A similar pathway has been described for *Nocardioides* sp. JS614; in this case the genes are chromosomal [6]. As shown in Scheme 1, the pathway begins with hydroxylation of the pyridyl ring of nicotine by the enzyme nicotine dehydrogenase to yield 6-hydroxynicotine [7]. Based on the gene sequence, this enzyme was identified as a member of the family of molybdopterin enzymes that also includes xanthine oxidoreductase and aldehyde oxidase [8]. Comparison of the pAO1 sequence with that of xanthine oxidoreductase identified *ndhs*, *ndhm*, and *ndhl* (initially designated *ndhABC*) as coding for three proteins: a 14.9 kDa subunit containing an iron–sulfur cluster, a 30 kDa subunit with an FAD binding site, and an 87.7 kDa subunit containing the molybdopterin site, respectively [9–10]. Consistent with this identification, expression of the active enzyme required molybdopterin [9], and pAO1 contains a number of genes that have been identified as coding for proteins involved in uptake of molybdenum and biosynthesis of the molybdopterin cofactor [11]. The mechanism of Scheme 2 can be written for nicotine dehydrogenase by analogy to the mechanism of xanthine oxidoreductase [8]. Here, the oxygen that is incorporated into the product initially comes from water, and the two electrons produced are transferred through the iron–sulfur subunit to the FAD and thence to the final electron acceptor.

**Figure 1:**
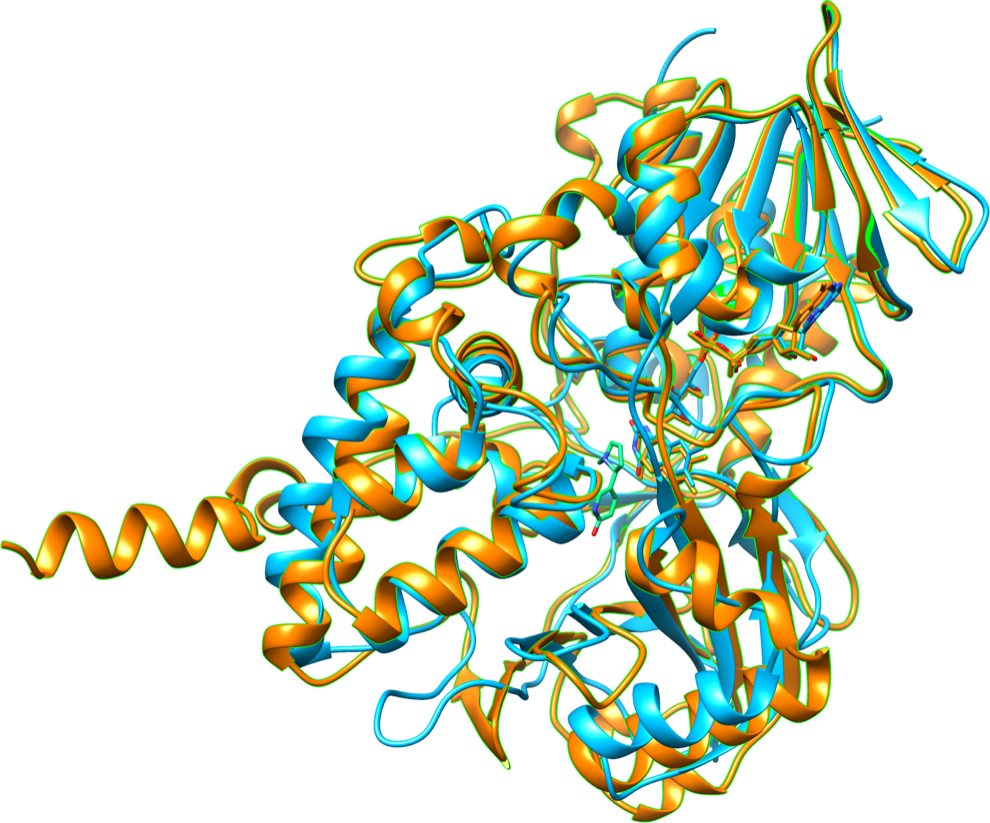
Overlay of the structure of LHNO (blue, pdb file 3NG7) with that of human MAO B (orange, pdb file 2FXU). The bound 6-hydroxynicotine is shown with green carbons.

**Scheme 3:**
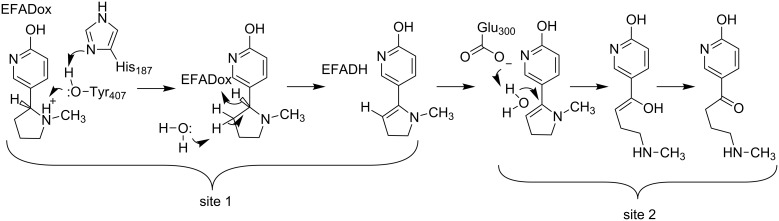
Proposed mechanism of LHNO [21].

**Scheme 4:**
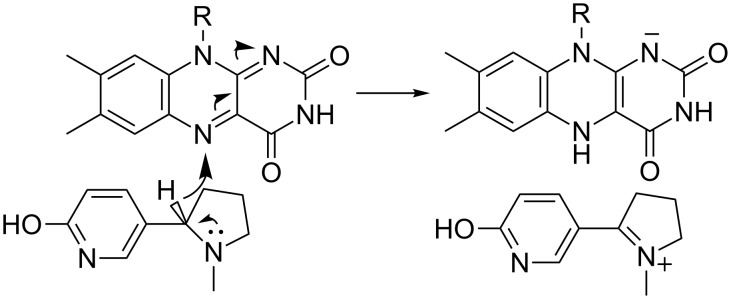
Mechanism of LHNO.

L-6-Hydroxynicotine oxidase (LHNO) catalyzes the subsequent oxidation of L-6-hydroxynicotine to 6-hydroxy-*N*-methylmysomine [12]. Purified LHNO contains non-covalently bound FAD [13], and the gene sequence is most similar to those of eukaryotic monoamine oxidases [14]. Several high-resolution structures of the enzyme from *A. nicotinovorans* are available, including substrate and product complexes [15]. These structures confirm that the protein is a member of the monoamine oxidase (MAO) family of flavoproteins (Figure 1) [16]. The reaction product was originally identified as arising from oxidation of the C2–C3 bond of the pyrrolidine ring [17]. Based on the structures and this product identification, a detailed mechanism was proposed in which initial oxidation of L-6-hydroxynicotine in the active site is followed by hydrolysis of the oxidized amine in a second site to yield 6-hydroxypseudooxynicotine (Scheme 3) [15]. However, a recent analysis of the structure of the product of the LHNO reaction utilizing NMR and continuous-flow mass spectrometry established that the enzyme catalyzes oxidation of the C2–N bond, not the C2–C3 bond, in line with the typical reactions catalyzed by members of the MAO family [18]. In addition, mutagenesis of His187, Glu300, and Tyr407 established that they are not involved in catalysis. Subsequent mechanistic studies of the reaction using pH and solvent isotope effects established that the reaction catalyzed by LHNO is the same as other flavin amine oxidases, direct hydride transfer from the uncharged amine to the flavin (Scheme 4) [19–20]. Hydrolysis to form 6-hydroxypseudooxynicotine occurs in solution after release of the oxidized amine from the enzyme.

**Figure 2:**
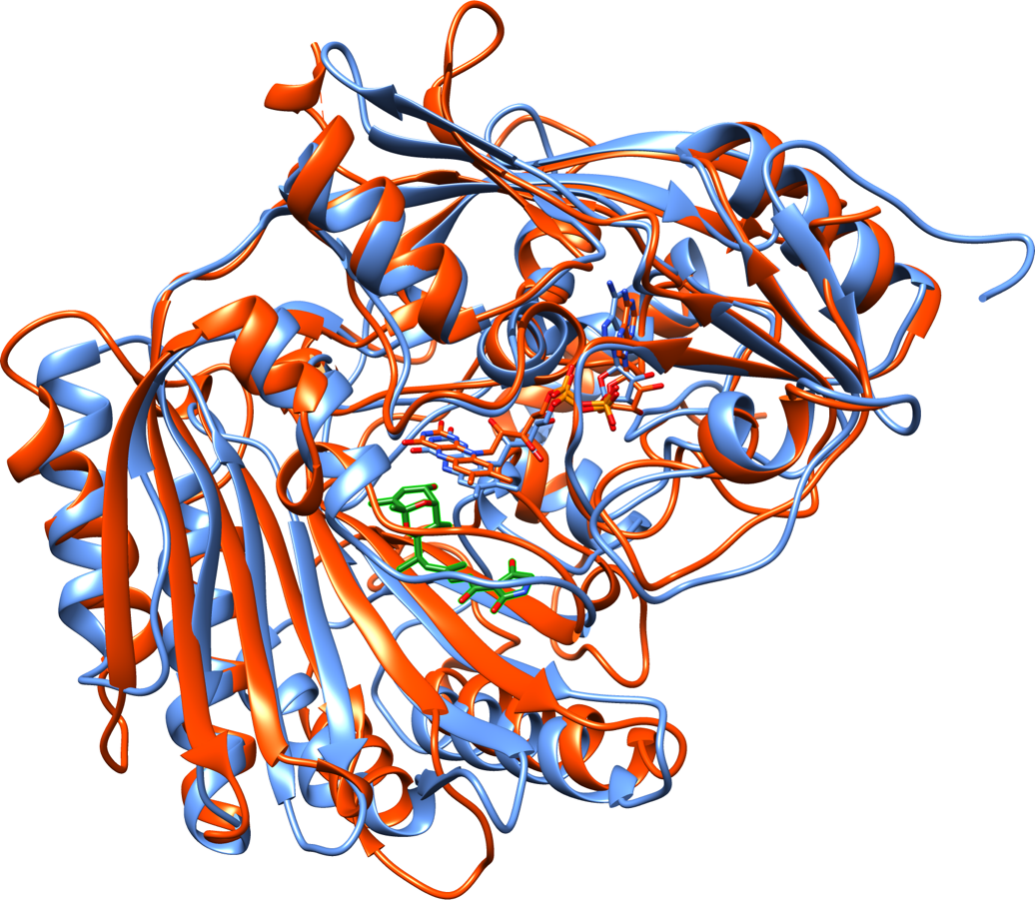
Overlay of the structures of DHNO (blue, pdb file 2bvf) and tirandamycin oxidase (orange, pdb file 2y3s), another member of the *p*-cresol methylhydroxylase/vanillyl oxidase family. The carbon atoms of tirandamycin are in green.

While the dominant form of nicotine found in tobacco is (*S*)-nicotine, the (*R*)-stereoisomer is also found at detectable levels [22]. Nicotine dehydrogenase is reported not to be stereospecific, in that it can catalyze the hydroxylation of (*R*)-nicotine to (*R*)-6-hydroxynicotine; thus, this enzyme is a likely candidate for the enzyme catalyzing the first step in the metabolism of both stereoisomers [23]. The subsequent step requires an additional enzyme. The pAO1 plasmid contains the gene for a D-6-hydroxynicotine oxidase (DHNO) in addition to that for LHNO. The product of the reaction catalyzed by DHNO is identical to that of the LHNO reaction, so that this enzyme was also initially identified as catalyzing the oxidation of the C2–C3 bond [17]. However, NMR analysis of the product has also recently established that DHNO catalyzes oxidation of the C2–N bond [24]. The sequence of DHNO from *A. nicotinovorans* identifies it as a member of the p-cresol methylhydroxylase/vanillyl oxidase family of flavoproteins [25]. As is common for members of this family, the FAD in DHNO is covalently bound to the protein, in this case through a C8α-histidyl linkage [26]. The subsequent determination of the crystal structure of the enzyme confirmed these conclusions (Figure 2) [27]. Docking of (*R*)-6-hydroxynicotine into the structure yielded a model for substrate binding.

**Scheme 5:**
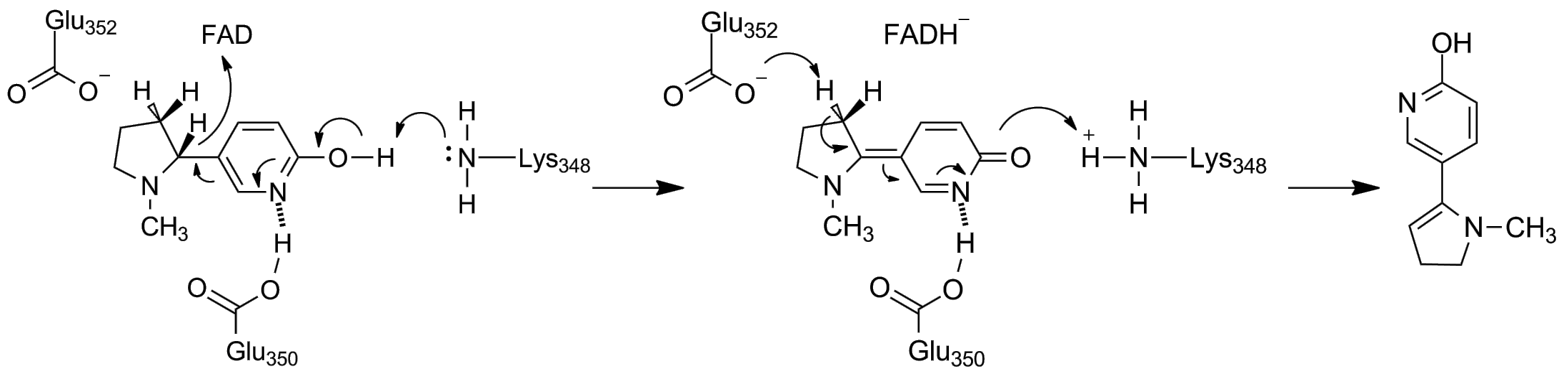
Proposed mechanism for DHNO [27].

Vanillyl oxidase catalyzes the oxidation of 4-hydroxybenzyl alcohols, the oxidative deamination of 4-hydroxybenzylamines, and the oxidative demethylation of 4-(methoxymethyl)phenols via a quinone methide intermediate [28–29]. Based on this precedent and the assumption that DHNO oxidizes the C2–C3 bond, Koetter and Schultz [27] proposed the mechanism shown in Scheme 5 for DHNO. However, members of the *p*-cresol methylhydroxylase/vanillyl oxidase family catalyze an extremely diverse set of reactions, including oxidation of non-aromatic alcohols and amines [30], and DALI [31] identifies several enzymes catalyzing oxidation of nonaromatic substrates as having similar structures to DHNO. Indeed, recent mechanistic studies of DHNO are more consistent with the simple mechanism of Scheme 4 (Fitzpatrick et al., manuscript in preparation). The proposed quinone methide is not detected during stopped-flow analyses of either the wild-type enzyme or the E352Q variant, (*R*)-6-chloronicotine and (*R*)-nicotine, which would not form the quinone methide, are still substrates, and there is no solvent isotope effect on amine oxidation. In addition, DHNO E350L/E352D has been developed as a reagent for stereospecific oxidation of a variety of (*R*)-amines, including a nicotine analog that does not contain an aromatic ring [24]. These results provide further evidence against the mechanism shown in Scheme 5 for DHNO.

Water reacts with the 6-hydroxy-*N*-methylmyosmine formed by either LHNO or DHNO to form 6-hydroxypseudooxynicotine in a reaction that appears to be non-enzymatic. 6-Hydroxypseudooxynicotine dehydrogenase (also known as ketone dehydrogenase [3]) then catalyzes the hydroxylation of the pyridyl ring of 6-hydroxypseudooxynicotine to form 2,6-dihydroxypseudooxynicotine [32]. Based on the sequence of pAO1, the enzyme was identified as a molybdopterin enzyme containing three subunits coded for by the *kdha*, *dhb* and *kdhc* genes [14]. The predicted sequences of Kdha and Kdhb show significant similarity to the small and medium subunits of nicotine dehydrogenase, while that of Kdhc shows the highest similarity to chicken xanthine dehydrogenase. The spectral properties of the partially purified protein are consistent with 6-hydroxypseudooxynicotine dehydrogenase being a molybdopterin protein, and the recombinant Kdhc (also known as KdhL) contains Mo and a cofactor derived from CTP [33]. While no mechanistic studies of the enzyme have been reported, its mechanism is likely to resemble those of nicotine dehydrogenase (Scheme 2) and other molybdopterin enzymes [8].

**Scheme 6:**
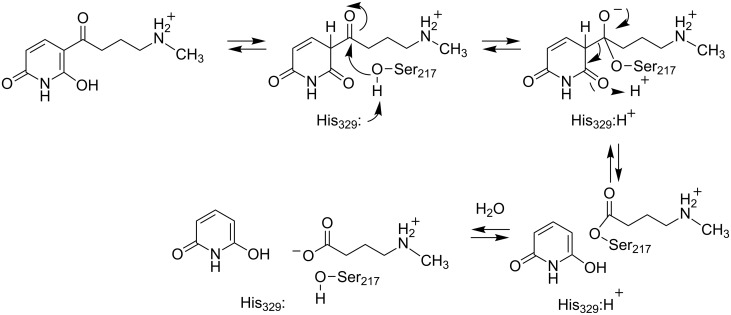
Mechanism of 2,6-dihydroxypseudooxynicotine hydrolase [37].

2,6-Dihydroxypseudooxynicotine hydrolase, the enzyme catalyzing the next step in the pyridine pathway, the cleavage of 2,6-dihydroxypseudooxynicotine to 2,6-dihydroxypyridine and *N*-methylaminobutyrate [34], was identified only after expression of a protein encoded by an open reading frame in pAO1 located next to the *kdhl* gene for the large subunit of 6-hydroxypseudooxynicotine dehydrogenase [35]. This protein was able to catalyze the cleavage of 2,6-dihydroxypseudooxynicotine without any added cofactors. A BLAST analysis of the sequence identified the protein as a member of the α/ß hydrolase family, which catalyzes a broad range of hydrolase and lyase reactions [36]. The subsequent determination of the crystal structure of the enzyme confirmed that it is an α/ß hydrolase, and mutagenesis identified the members of the catalytic triad as His_329_, Ser_217_, and Asp_300_ [37]. By analogy to other members of the family, the mechanism shown in Scheme 6 was proposed. There is an initial tautomerization to the diketo form of the substrate; Glu_248_ acts as both the initial proton acceptor and subsequent proton for this reaction. Nucleophilic attack of Ser_217_ on the substrate carbonyl followed by collapse of the tetrahedral intermediate generates an acyl–enzyme intermediate and the 2,6-dihydroxypyridine product. The subsequent hydrolysis of the acyl–enzyme intermediate then yields the *N*-methylaminobutyrate product. Other than the preliminary characterization of site-directed mutants of the protein, no mechanistic studies have been reported.

Two pathways have been identified for further metabolism of *N*-methylaminobutyrate. pAO1 contains separate genes for enzymes catalyzing the oxidative demethylation of *N*-methylaminobutyrate to form 4-aminobutyrate, *mabo*, and its oxidative deamination to form succinate semialdehyde and methylamine, *mao*. The expression of both proteins is regulated by nicotine [38–39], suggesting that both contribute in vivo. Mabo (γ-*N*-methylaminobutyrate demethylating oxidase) is similar in sequence to sarcosine dehydrogenase, and characterization of purified Mabo showed that it contains covalently-bound FAD and produces hydrogen peroxide as a product in addition to 4-aminobutryate [5,38]. Mabo also catalyzes the oxidative demethylation of sarcosine. Based on these results, the mechanism of the enzyme is similar to that of sarcosine oxidase, direct oxidation of the C–N bond of the substrate methyl group by hydride transfer [40]. The resulting 4-aminobutryrate is likely a substrate for a chromosomally-encoded aminotransferase, producing α-ketoglutarate and succinate semialdehyde. Mao (γ-*N*-methylaminobutyrate oxidase) contains noncovalently-bound flavin and catalyzes the oxidation of the other C–N bond of the methyl group in *N*-methylaminobutyrate to form methylamine and succinate semialdehyde, an MAO reaction [39]. While the *k*_cat_/*K*_m_ value of Mao with 4-aminobutyrate is only 8% that of Mabo, *A. nicotinovorans* grown on [^14^C]-nicotine produce [^14^C]-methylamine, suggesting that Mao operates in vivo. Finally, pAO1 also contains the *sad* gene that codes for an NADP^+^-dependent succinate semialdehyde dehydrogenase forming succinate as product [39].

**Figure 3:**
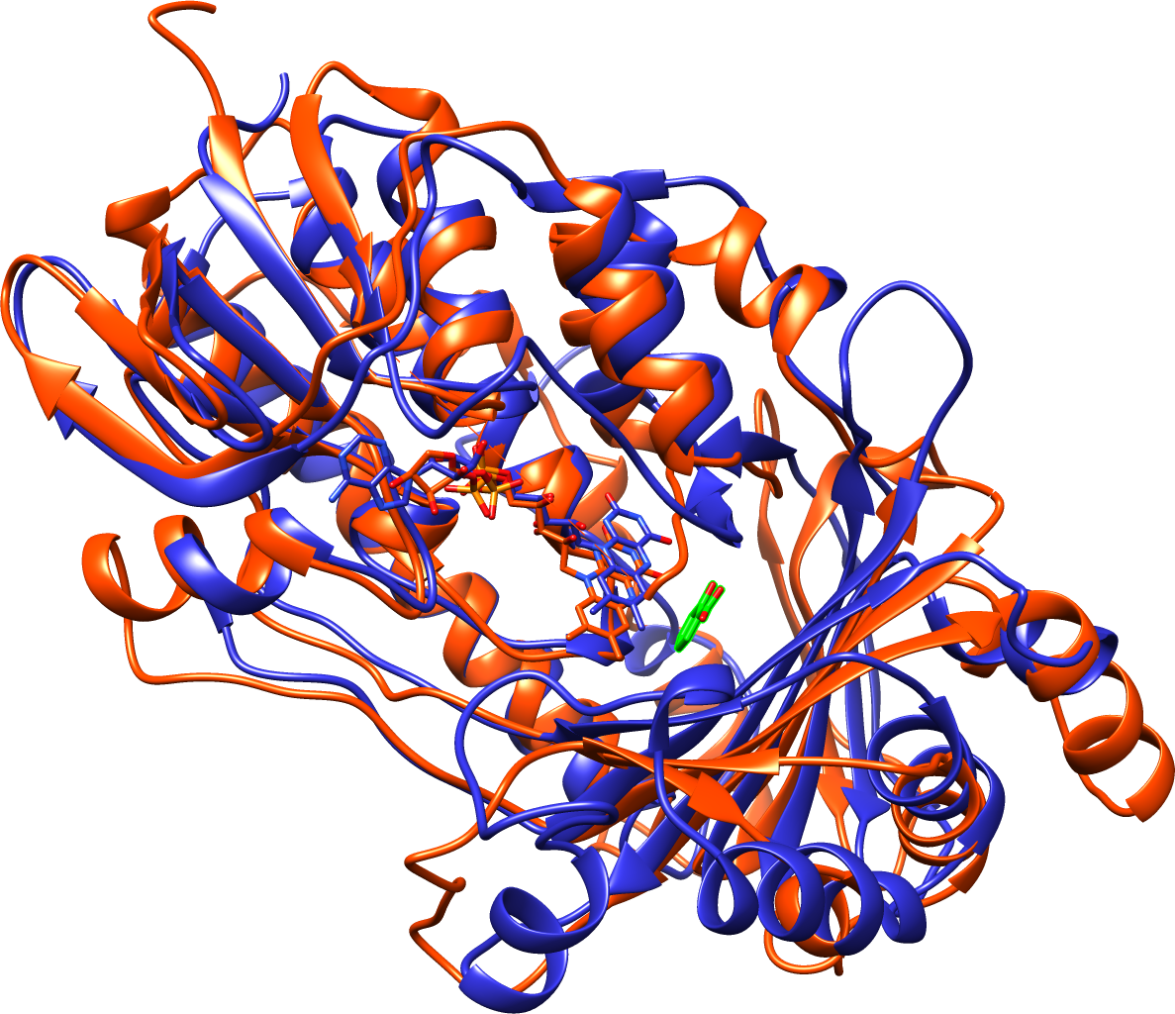
Overlay of structures of salicylate hydroxylase (orange, pdb file 5evy) and 2,3-dihydroxypyridine 3-hydroxylase (blue, pdb file 2vou). The salicylate bound to the latter is shown in green.

**Scheme 7:**
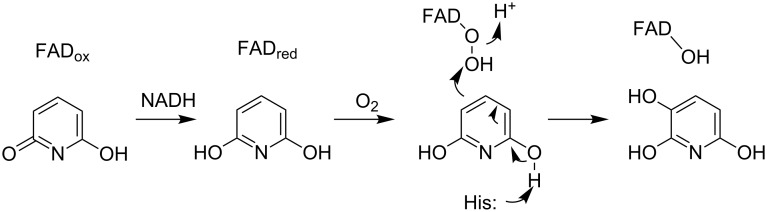
Mechanism of 2,3-dihydroxypyridine 3-hydroxylase [42].

2,3-Dihydroxypyridine 3-hydroxylase (2,6-DHPH), the enzyme converting 2,6-dihydroxypyridine to 2,3,6-trihydroxypyridine, has been cloned and characterized [10]. DHPH contains FAD and requires NADH and oxygen [41], and the sequence of the protein is similar to that of salicylate hydroxylase, although the sequence identity is only 21%. This allowed identification of the enzyme as a flavin-dependent phenol hydroxylase, a conclusion that was subsequently confirmed by the crystal structure of the enzyme (Figure 3) [42]. Based on the mechanism of this family of enzymes [43], the likely mechanism for this enzyme is as shown in Scheme 7. Flavin reduction by NADH is followed by the formation of the peroxyflavin hydroxylating intermediate. Attack of the substrate, activated by deprotonation of a substrate hydroxy group, on the peroxyflavin yields the hydroxylated product after a tautomerization. Two histidyl residues have been proposed to be involved in accepting the substrate proton. The details of further catabolism of 2,3,6-trihydroxypyridine are unclear. The compound can oxidatively dimerize to form nicotine blue [44], which is secreted into the medium. However, this has been proposed to be a byproduct, with the major pathway involving formation of maleamate, maleate, and fumarate [45].

### The pyrrolidine pathway

**Scheme 8:**
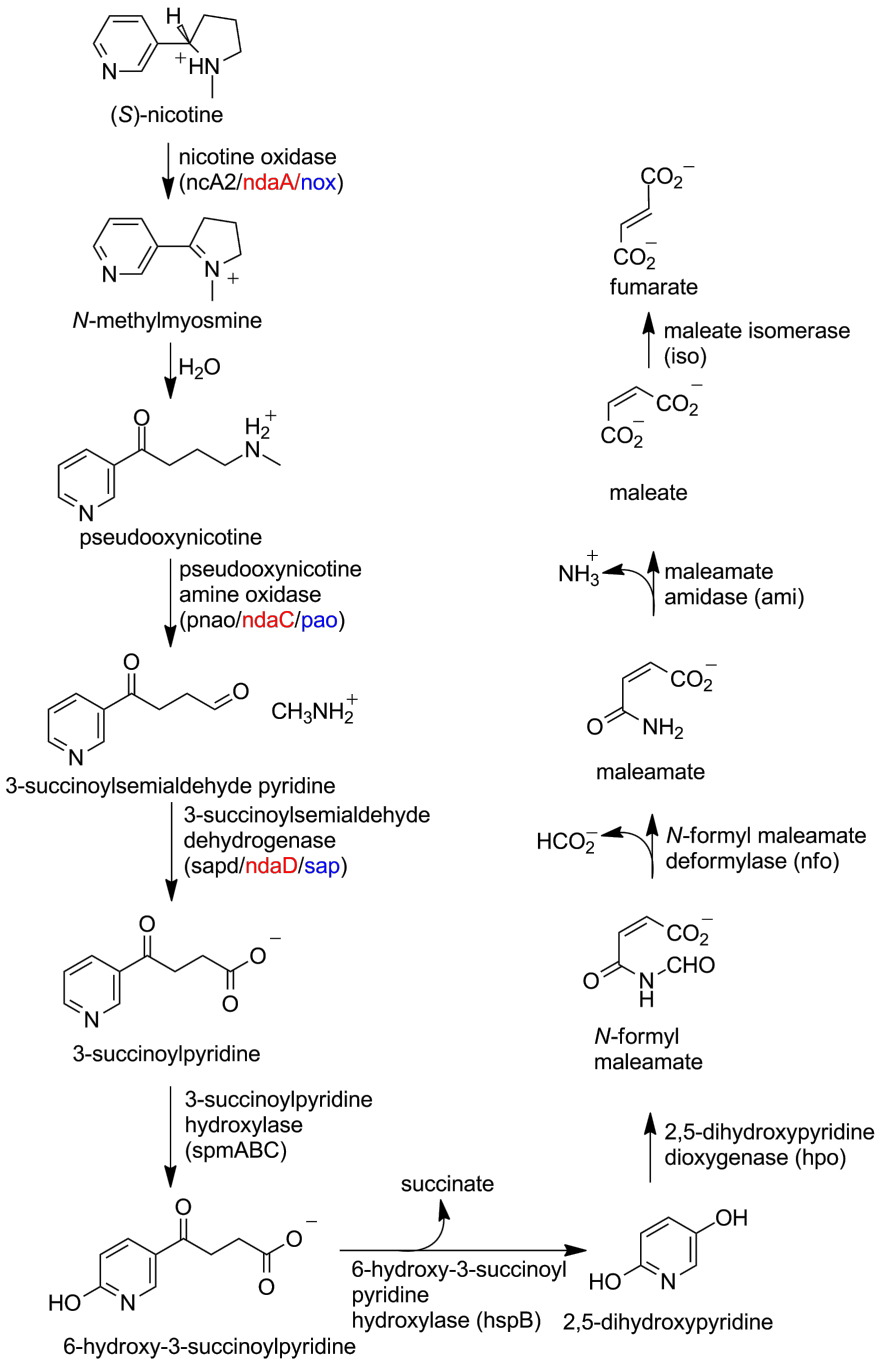
The pyrrolidine pathway for nicotine degradation by pseudomonads. The gene names for *P. putida* S16 (black), *P. putida* J5 (red), and *Pseudomonas* sp. HZN6 (blue) are in parentheses.

**Figure 4:**
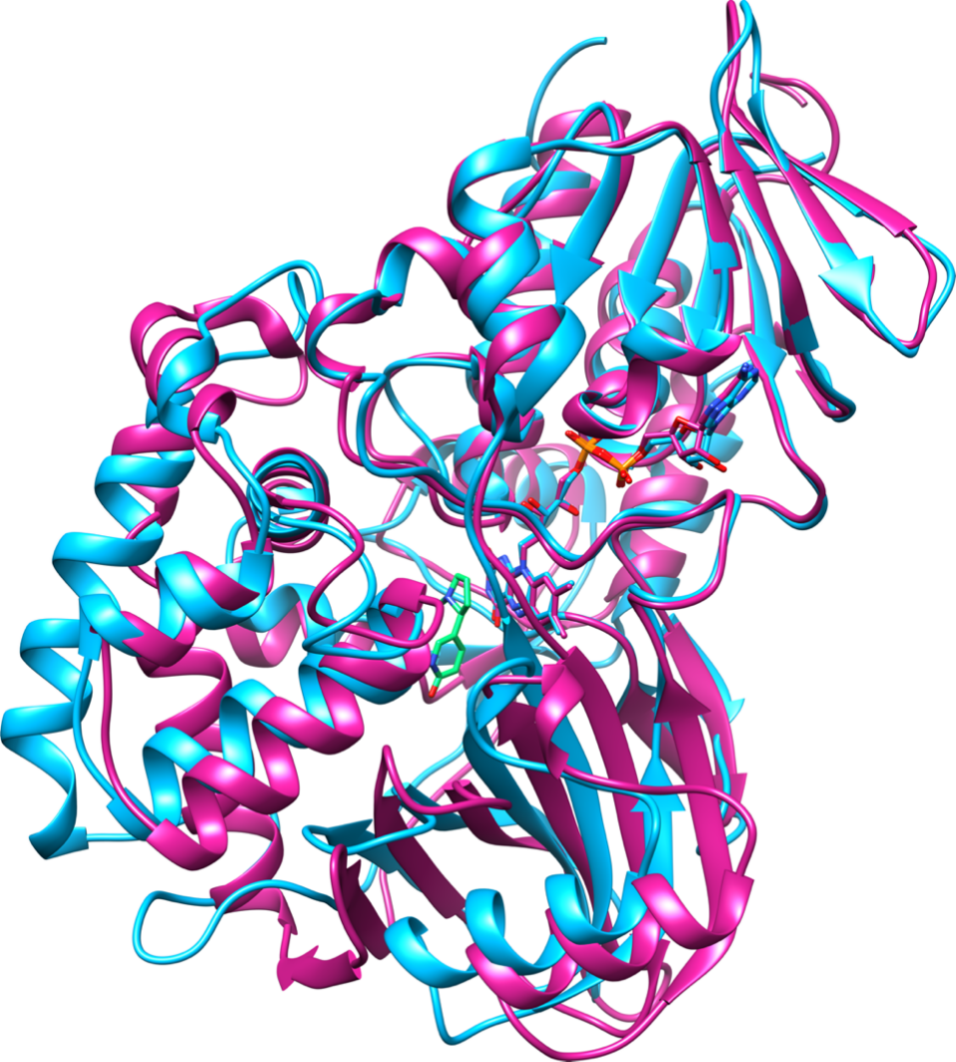
Overlay of the structure of LHNO (magenta, pdb file 3NG7) with that of NicA2 (magenta, pdb file 5ttj) B (green, pdb file 2FXU). The bound 6-hydroxynicotine is shown with green carbons.

The metabolic pathway for nicotine degradation found in a number of pseudomonads (Scheme 8) [46–50] has been described as the pyrrolidine pathway. The initial oxidation of the pyrrolidine ring is catalyzed by the enzyme nicotine oxidase. In *P. putida* S16, the *nicA1* and *nicA2* genes produce separate enzymes that are both reported to have the ability to catalyze this reaction [48]. NicA1 was also reported to catalyze the subsequent oxidation of pseudooxynicotine to 3-succinylpyridine and methylamine, but no kinetic parameters for the two reactions were reported [51]. However, deletion of *nicA2* but not of *nicA1* prevents *P. putida* S16 from degrading nicotine, making it most likely that NicA2 is the relevant nicotine oxidase for this pathway [48]. In addition the amino acid sequence of NicA1 has no similarities to bacterial oxidases or dehydrogenases, instead resembling components of the bacterial electron transport chain. Thus, the function of NicA1 remains unclear, and NicA2 is likely the true nicotine oxidase. The *ndaA* gene in *P. putida* J5, required for degradation of nicotine by that organism, codes for a protein that is 99% identical in sequence to that of NicA2 [52], so that NdaA is also likely to be a nicotine oxidase. The structure of NicA2 was recently determined, showing that the protein is a member of the MAO family with the same overall structure as LHNO (Figure 4) [53]. As is the case with LHNO, the NicA2-catalyzed reaction has generally been accepted to involve oxidation of a carbon–carbon bond in (*S*)-nicotine to form *N*-methylmyosmine. The recent evidence that the product of the oxidation of 6-hydroxynicotine by LHNO and DHNO arises from oxidation of a carbon–nitrogen bond [18,24] and the similarity of the active sites of LHNO and NicA2 to that of MAO makes it much more likely that the NicA2 instead catalyzes oxidation of the substrate carbon–nitrogen bond as shown in Scheme 4.

Cloning and expression of the protein encoded by the *nox* gene of *Pseudomonas sp.* HZN6 showed that it also catalyzes oxidation of nicotine to pseudooxynicotine [54]. The sequence of the protein is most similar to that of LHNO and several members of the MAO family, consistent with Nox being a nicotine oxidase similar to NicA2. Nox is reported to be able to oxidize both stereoisomers of nicotine equally well, in contrast to the stereospecificity of LHNO and DHNO.

**Scheme 9:**
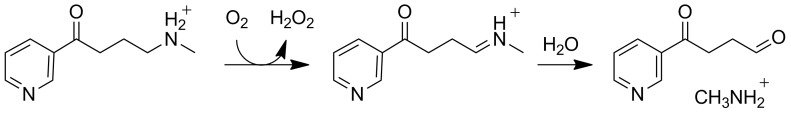
The pseudooxynicotine amine oxidase reaction.

Pseudooxynicotine amine oxidase, the enzyme catalyzing the next step in the pathway, has been characterized from both *P. putida* S16 (Pnao) [48,55] and *P. putida* HZN6 (Pao) [47]. Both are FAD-containing enzymes whose sequences place them in the MAO family of flavoproteins. The sequence of NdaC from *P. putida* J5 is identical to that of Pnao, although the protein itself has not been characterized, and loss of *ndaC* eliminates the ability of cells to metabolize pseudooxynicotine [52]. In the case of Pnao the source of the oxygen in the 3-succinoylsemialdehyde pyridine product has been shown to be water [55], establishing the reaction catalyzed by the enzyme as shown in Scheme 9, with the hydrolytic step being nonenzymatic. This is essentially the same reaction as that catalyzed by *A. nicotinovorans* γ-*N*-methylaminobutyrate demethylating oxidase (Mabo).

*E. coli* expressing the *sap* gene from *Pseudomonas sp.* HZN6 will catalyze the NADP^+^-dependent oxidation of 3-succinoylsemialdehyde pyridine to 3-succinoylpyridine [47], making SAP the likely 3-succinoylsemialdehyde dehydrogenase in the pyrrolidine pathway. The sequence of the enzyme identifies it as an aldehyde dehydrogenase [56], but the protein itself does not appear to have been characterized. In *P. putida* S16 and J5, sequence analyses have identified Spad and ndaD, respectively, as the likely 3-succinoylsemialdehyde pyridine dehydrogenases, and ndaD is required for *P. putida* J5 to convert 3-succinoylsemialdehyde pyridine to 3-succinoylpyridine [48,52].

Growth of *P. putida* S16 on nicotine results in increased expression of NicA2, Pnao, Sapd, SpmABC, and HspB, but not NicA1 or HspA [48]. The sequences of SpmA, SpmB, and SpmC are similar to those of nicotine dehydrogenase and other members of the xanthine dehydrogenase family. In addition, disrupting *spma* prevents *P. putida* S16 from converting 3-succinoylpyrimidine to 6-hydroxy-3-succinoylpyridine [48]. These results support the identification of SpmABC as a molybdopterin enzyme that catalyzes this step in the pathway. Enzymes with this activity do not appear to have been identified as yet for *P. putida* S5 and *Pseudomonas sp.* HZN6.

**Scheme 10:**
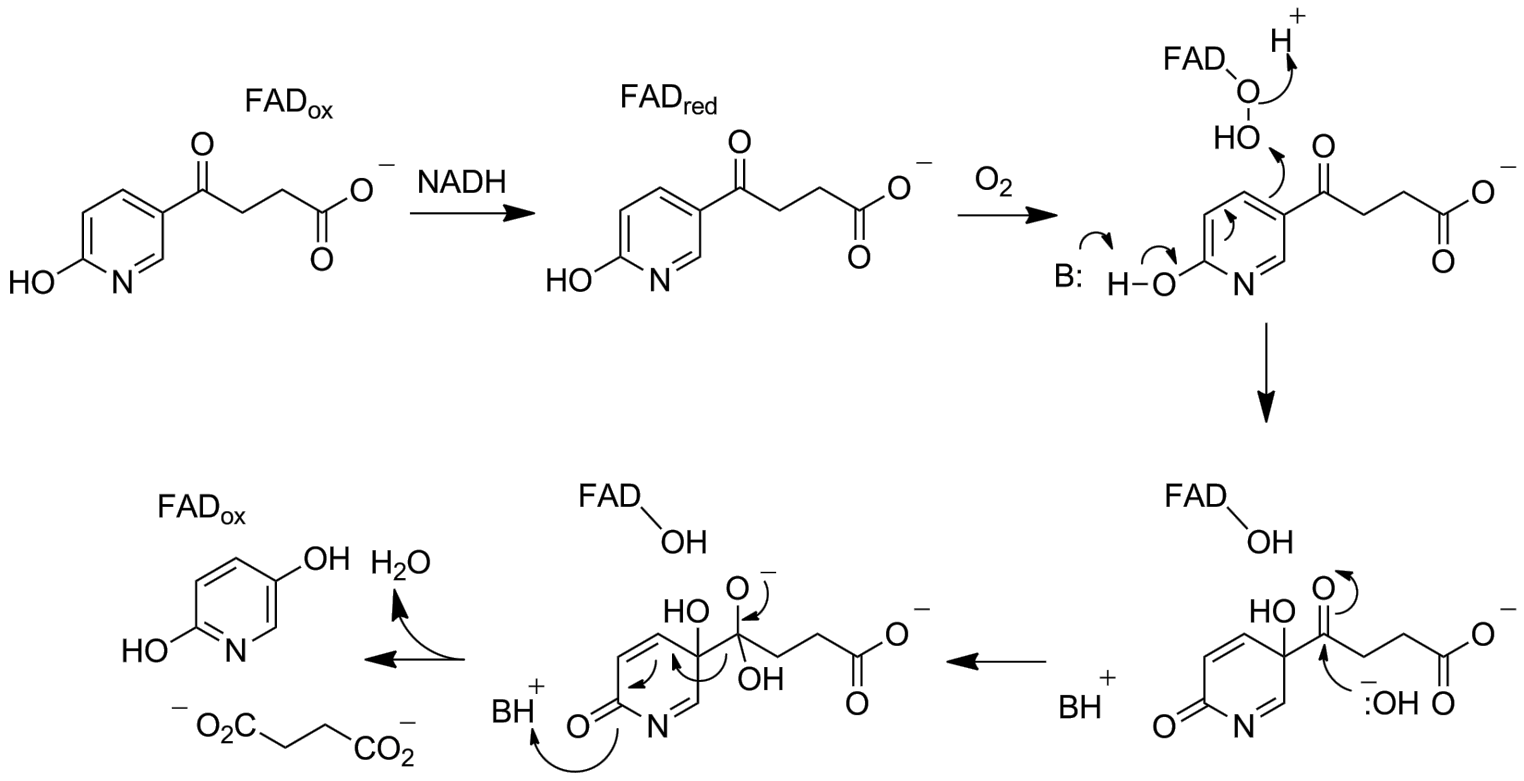
Mechanism of HspB [59].

HspA in *P. putida* S16 was originally identified as a 6-hydroxy-3-succinoylpyridine hydroxylase catalyzing the formation of 2,5-dihydroxypyridine from 6-hydroxy-3-succinoylpyridine based on the location of the gene in a gene cluster that conferred on *E. coli* the ability to degrade nicotine to 2,5-dihydroxypyridine [57]. The sequence of the protein is not similar to that of any proteins with known functions. Purified recombinant HspA was reported to require NADH to catalyze the cleavage of 6-hydroxy-3-succinoylpyridine, but detailed kinetic analyses were not done. However, levels of HspA do not increase when *P. putida* S16 is grown on nicotine, while levels of HspB do [48]. Subsequent analysis of recombinant HspB showed that it contains FAD and catalyzes the NADH-dependent conversion of 6-hydroxy-3-succinoylpyridine to 2,5-dihydroxypyridine [58–59]. The new oxygen atom in 2,5-dihydroxypyridine comes from O_2_, while that in succinate comes from H_2_O. The sequence of HspB is closest to those of a number of FAD-dependent hydroxylases, and a peroxyflavin was detected in stopped-flow analyses of the enzyme-catalyzed reaction. These results led to the mechanism shown in Scheme 10 for HspB. A similar enzyme has been isolated from *Pseudomonas* sp. ZZ-5 [60].

The subsequent steps in metabolism of 2,5-dihydropyridine by *P. putida* S16 were identified when the gene cluster *nic2* that contained *hspb* was sequenced, with the demonstration that incorporation of *nic2* into *E. coli* allowed cells to convert 6-hydroxy-3-succinoylpyridine to fumarate [61]. In addition to hspB and an unidentified open reading frame, four genes could be identified by sequence analyses as likely to code for proteins catalyzing the final steps in nicotine catabolism. These four proteins were expressed individually in *E. coli* and characterized. Hpo catalyzes the Fe(II)-dependent formation of *N*-formylmaleamate from 2,5-dihydroxypyridine in the absence of other cofactors or substrates; it was designated DHP dioxygenase. Both oxygen atoms in the product come from O_2_ and mutagenesis of the predicted iron ligand His257, His310, or Asp312 results in loss of activity, consistent with Hpo being a non-heme Fe(II)-dependent dioxygenase [62]. Nfo catalyzes the formation of maleamate from *N*-formylmaleamate and was designated *N*-formylmaleamate deformylase; its sequence identifies it as a member of the α/ß hydrolase superfamily [36]. Ami is a maleamate amidase that catalyzes the hydrolysis of maleamate to maleic acid plus ammonium; it also belongs to the α/ß hydrolase superfamily. Finally, Iso catalyzes the reversible isomerization of maleate to fumarate. Orthologues of all four of these enzymes have been identified as being involved in the metabolism of nicotinic acid by *P. putida* KT25440, which begins with the hydroxylation of nicotinic acid by the molybdopterin enzyme NicAB to form 6-hydroxynicotinic acid and its subsequent conversion to 2,5-dihydroxypyridine by the NADH- and FAD-dependent hydroxylase NicC [63–64].

### The hybrid pathway

**Scheme 11:**
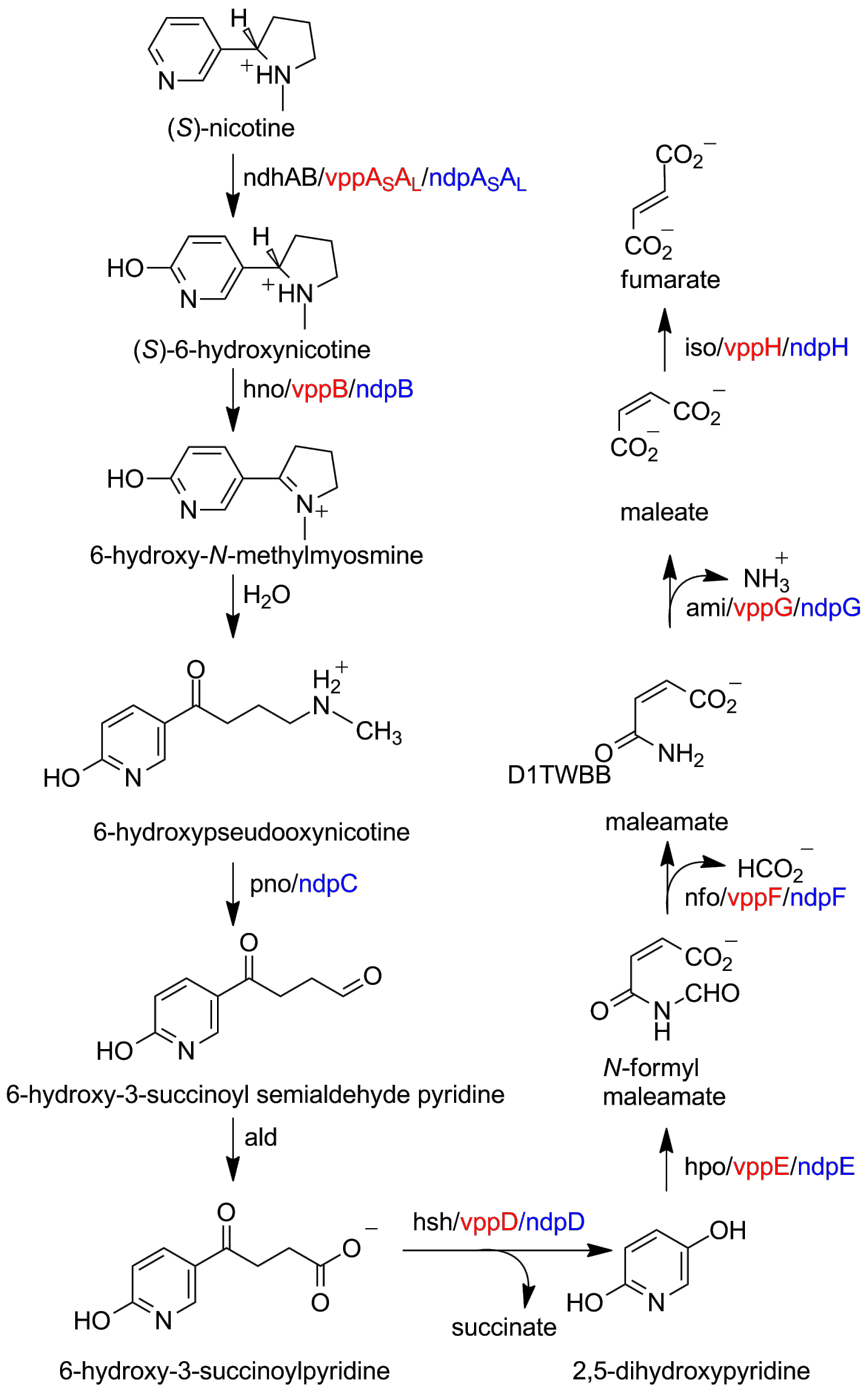
Hybrid pyridine/pyrrolidine pathway for nicotine metabolism in *Agrobacter tumefaciens* S33 (black), *Ochrobactrum* sp. SJY1 (red), and *Sphingomonas melonis* Ty (blue).

While the pyridine and pyrrolidine pathways are the best understand reactions by which bacteria degrade nicotine, additional pathways continue to be discovered. The best-characterized is a hybrid of the pyridine and pyrrolidine pathways (Scheme 11). Based on phylogenetic analysis, the pathway is more closely related to the pyrrolidine pathway, with both found predominantly in Gram-negative bacteria [65]. This pathway is best characterized for *Agrobacter tumefaciens* S33, *Ochrobactrum* sp. SJY1, *Sphingomonas melonis* TY, and *Shinella* sp. HZN7, but has been identified in other bacteria as well [65–70]. The pathway begins with the hydroxylation of nicotine, as in the pyridine pathway, but diverges after the formation of 6-hydroxypseudooxynicotine. The oxidative deamination of 6-hydroxypseudooxynicotine yields 6-hydroxy-3-succinoylsemialdehyde pyridine, an intermediate that is not present in the other two pathways; its oxidation forms 6-hydroxy-3-succinoylpyridine, which is processed further as in the pyrrolidine pathway. Elucidation of the hybrid pathway has relied on identification of intermediates and on comparison of gene sequences with those coding for enzymes known to be involved in nicotine catabolism in the pyridine and pyrrolidine pathways.

The identification of 6-hydroxy-L-nicotine, 6-hydroxy-*N*-methylmyosmine, 6-hydroxypseudooxynicotine, 6-hydroxy-3-succinoylpyridine, and 2,5-dihydroxypyridine as metabolites in cells of *A. tumefaciens* S33 degrading nicotine provided the initial evidence for a pathway different from those shown in Scheme 1 and Scheme 8 [66]. The complete genome of *A. tumefaciens* S33 was recently sequenced, allowing identification of candidate genes for all of the steps for the hybrid pathway for nicotine degradation in that organism (Scheme 11) [69]. The nicotine dehydrogenase Ndh and the 6-hydroxypseudooxynicotine oxidase Pno, one of the two novel enzymes in this pathway, have both been purified and characterized; they are reported to form a complex [71]. The sequences of the two enzymes identify Ndo as a member of the family of molybdopterin enzymes such as xanthine dehydrogenase and Pno as a member of the trimethylamine dehydrogenase family of flavoproteins [72]. Consistent with this identification, purified Pno contains FMN and a 4Fe/4S center. Preliminary kinetics have been reported for both enzymes. 6-LHNO activity has been detected in crude cell lysates of *A. tumefaciens* S33 grown on nicotine, but the pure enzyme has not been described [66]. The NADH-dependent 6-hydroxy-3-succinoylpyridine hydroxylase Hsh has been partially purified from this organism; this enzyme is likely an FAD-dependent hydroxylase similar to HspB, but succinate has not been shown to be a product in this case [66].

6-Hydroxy-L-nicotine, 6-hydroxy-*N*-methylmyosmine, 6-hydroxypseudooxynicotine, 6-hydroxy-3-succinoylpyridine, and 2,5-dihydroxypyridine have also been isolated from cells of *Ochrobactrum* sp. SJY1 growing on nicotine [68]. The sequencing of its genome allowed identification of several of the genes involved in nicotine degradation (Scheme 11) [73]. VppB, VppD, and VppE have all been expressed in recombinant form [68]. VppB is a flavin amine oxidase that catalyzes the oxidation of 6-hydroxynicotine, establishing it as an LHNO, although the sequence of the protein is closer to that of *P. putida* S16 NicA2 than LHNO from *A. nicotinovorans*. VppD is an NAD(P)-dependent flavin monooxygenase whose sequence is 62% identical to that of *P. putida* S16 HspB. The crude recombinant VppE catalyzes the iron-dependent oxidation of 2,5-dihydroxypyridine to *N*-formylmaleamate, the same reaction as is catalyzed by the dioxygenase Hpo from *P. putida* S16.

In *Sphingomonas melonis* TY, the genes for the metabolism of nicotine are found in the *ndp* gene cluster [65]. The mRNA levels for *ndpA-H* all increase 10 to 100-fold upon growth in the presence of nicotine. Sequence similarities of >35% in all cases to genes in *P. putida* S16 involved in nicotine metabolism suggested that the roles of each are as those shown in Scheme 11. NdpA-D were all expressed in recombinant form and shown to confer on cells the ability to catalyze the proposed reactions, confirming the identification of NdpA as a nicotine dehydrogenase, NdpB as an LHNO, NdpC as an oxidative demethylase, and NdpD as 6-hydroxy-3-succinoylpyridine 3-monooxygenase. The enzyme catalyzing formation of 6-hydroxy-3-succinoylpyridine from the aldehyde was not identified in the *ndp* cluster; this activity was attributed to a non-specific semialdehyde dehydrogenase.

Characterization of the enzymes involved in the hybrid pathway in *Shinella* sp. HZN7 is less complete. 6-Hydroxynicotine, 6-hydroxy-*N*-methylmyosmine, 6-hydroxypseudooxynicotine, 6-hydroxy-3-succinoylpyridine, and 2,5-dihydroxypyridine have been confirmed as intermediates in the degradation of nicotine by this organism [67]. This bacterium is also able to utilize 2,5-dihydroxypyridine as a sole carbon source, establishing the presence of the complete pathway. 6-Hydroxy-3-succinoyl semialdehyde pyridine was not reported as a detectable intermediate, but it might not have accumulated to sufficient levels for detection. The genes *nctA1* and *nctA2* code for proteins with sequences identical to the nicotine hydroxylase VppAB from *Ochrobactrum* sp. SJY1 [74]. The gene *nctB* was identified as required for nicotine degradation using genetic approaches; NctB was expressed in *E. coli* and the purified protein shown to be an LHNO similar to the enzyme from *A. nicotinovorans* in its kinetic properties [18,75]. The genes responsible for the other enzymes were tentatively identified by comparison with the sequences of the enzymes from *Ochrobactrum* sp. SJY1 and *A. tumefaciens* when the complete genome of *Shinella* sp. HZN7 was sequenced [76].

## Conclusion

This review has attempted to summarize our present understanding of the microbial metabolism of nicotine, with an emphasis on the enzymes involved. It has not attempted to address the less understood fungal metabolism of nicotine. Elucidation of the details of nicotine metabolism remains one of intense investigation, and the rapid increase in genomic sequences means that additional organisms capable of degrading nicotine are frequently being described. Many of the enzymes involved are poorly characterized even if mechanisms can be proposed for them based on their homology to known families of enzymes, and not all of the enzymes have been identified in some cases. Still, these enzymes are already being used to produce new synthetically catalysts, while the pathways are being retooled to produce useful synthetic intermediates.

## References

[1] Liu J, Ma G, Chen T, Hou Y, Yang S, Zhang K-Q, Yang J (2015). Appl Microbiol Biotechnol.

[2] Yu W, Wang R, Li H, Liang J, Wang Y, Huang H, Xie H, Wang S (2017). Biotechnol Biofuels.

[3] Brandsch R (2006). Appl Microbiol Biotechnol.

[4] Brandsch R, Hinkkanen A E, Decker K (1982). Arch Microbiol.

[5] Igloi G L, Brandsch R (2003). J Bacteriol.

[6] Ganas P, Sachelaru P, Mihasan M, Igloi G L, Brandsch R (2008). Arch Microbiol.

[7] Hochstein L I, Rittenberg S C (1959). J Biol Chem.

[8] Hille R, Hall J, Basu P (2014). Chem Rev.

[9] Grether-Beck S, Igloi G L, Pust S, Schilz E, Decker K, Brandsch R (1994). Mol Microbiol.

[10] Baitsch D, Sandu C, Brandsch R, Igloi G L (2001). J Bacteriol.

[11] Menéndez C, Otto A, Igloi G, Nick P, Brandsch R, Schubach B, Böttcher B, Brandsch R (1997). Eur J Biochem.

[12] Gries F A, Decker K, Brühmueller M (1961). Hoppe-Seyler's Z Physiol Chem.

[13] Dang Dai V, Decker K, Sund H (1968). Eur J Biochem.

[14] Schenk S, Hoelz A, Krauß B, Decker K (1998). J Mol Biol.

[15] Kachalova G, Decker K, Holt A, Bartunik H D (2011). Proc Natl Acad Sci U S A.

[16] Gaweska H, Fitzpatrick P F (2011). Biomol Concepts.

[17] Decker K, Dai V D (1967). Eur J Biochem.

[18] Fitzpatrick P F, Chadegani F, Zhang S, Roberts K M, Hinck C S (2016). Biochemistry.

[19] Fitzpatrick P F, Chadegani F, Zhang S, Dougherty V (2017). Biochemistry.

[20] Fitzpatrick P F (2010). Arch Biochem Biophys.

[21] Kachalova G S, Bourenkov G P, Mengesdorf T, Schenk S, Maun H R, Burghammer M, Riekel C, Decker K, Bartunik H D (2010). J Mol Biol.

[22] Armstrong D W, Wang X, Ercal N (1998). Chirality.

[23] Decker K, Eberwein H, Gries F A, Bruehmueller M (1961). Biochem Z.

[24] Heath R S, Pontini M, Bechi B, Turner N J (2014). ChemCatChem.

[25] Fraaije M W, van Berkel W J H, Benen J A E, Visser J, Mattevi A (1998). Trends Biochem Sci.

[26] Möhler H, Brühmüller M, Decker K (1972). Eur J Biochem.

[27] Koetter J W A, Schulz G E (2005). J Mol Biol.

[28] Fraaije M W, van Berkel W J H (1997). J Biol Chem.

[29] Fraaije M W, Veeger C, Van Berkel W J H (1995). Eur J Biochem.

[30] Ewing T A, Fraaije M W, Mattevi A, van Berkel W J H (2017). Arch Biochem Biophys.

[31] Holm L, Rosenström P (2010). Nucleic Acids Res.

[32] Richardson S H, Rittenberg S C (1961). J Biol Chem.

[33] Sachelaru P, Schiltz E, Brandsch R (2006). Appl Environ Microbiol.

[34] Gherna R L, Richardson S H, Rittenberg S C (1965). J Biol Chem.

[35] Sachelaru P, Schiltz E, Igloi G L, Brandsch R (2005). J Bacteriol.

[36] Rauwerdink A, Kazlauskas R J (2015). ACS Catal.

[37] Schleberger C, Sachelaru P, Brandsch R, Schulz G E (2007). J Mol Biol.

[38] Chiribau C B, Sandu C, Fraaije M, Schiltz E, Brandsch R (2004). Eur J Biochem.

[39] Chiribau C-B, Mihasan M, Ganas P, Igloi G L, Artenie V, Brandsch R (2006). FEBS J.

[40] Ralph E C, Hirschi J S, Anderson M A, Cleland W W, Singleton D A, Fitzpatrick P F (2007). Biochemistry.

[41] Holmes P E, Rittenberg S C (1972). J Biol Chem.

[42] Treiber N, Schulz G E (2008). J Mol Biol.

[43] Huijbers M M, Montersino S, Westphal A H, Tischler D, van Berkel W J H (2014). Arch Biochem Biophys.

[44] Knackmuss H-J, Beckmann W (1973). Arch Mikrobiol.

[45] Kaiser J P, Feng Y, Bollag J M (1996). Microbiol Rev.

[46] Wang S N, Liu Z, Tang H Z, Meng J, Xu P (2007). Microbiology (London, U K).

[47] Qiu J, Ma Y, Wen Y, Chen L, Wu L, Liu W (2012). Appl Environ Microbiol.

[48] Tang H, Wang L, Wang W, Yu H, Zhang K, Yao Y, Xu P (2013). PLoS Genet.

[49] Raman G, Sakthivel N, Park S (2015). Genome Announce.

[50] Li A, Qiu J, Chen D, Ye J, Wang Y, Tong L, Jiang J, Chen J (2017). Mar Drugs.

[51] Tang H, Wang L, Meng X, Ma L, Wang S, He X, Wu G, Xu P (2009). Appl Environ Microbiol.

[52] Xia Z, Zhang W, Lei L, Liu X, Wei H-L (2015). Appl Microbiol Biotechnol.

[53] Tararina M A, Janda K D, Allen K N (2016). Biochemistry.

[54] Qiu J, Ma Y, Zhang J, Wen Y, Liu W (2013). Appl Environ Microbiol.

[55] Hu H, Wang W, Tang H, Xu P (2015). Sci Rep.

[56] Liu Z-J, Sun Y-J, Rose J, Chung Y-J, Hsiao C-D, Chang W-R, Kuo I, Perozich J, Lindahl R, Hempel J (1997). Nat Struct Biol.

[57] Tang H, Wang S, Ma L, Meng X, Deng Z, Zhang D, Ma C, Xu P (2008). Appl Environ Microbiol.

[58] Tang H, Yao Y, Zhang D, Meng X, Wang L, Yu H, Ma L, Xu P (2011). J Biol Chem.

[59] Yu H, Hausinger R P, Tang H-Z, Xu P (2014). J Biol Chem.

[60] Wei T, Zang J, Zheng Y, Tang H, Huang S, Mao D (2017). Catalysts.

[61] Tang H, Yao Y, Wang L, Yu H, Ren Y, Wu G, Xu P (2012). Sci Rep.

[62] Kovaleva E G, Lipscomb J D (2008). Nat Chem Biol.

[63] Jiménez J I, Canales Á, Jiménez-Barbero J, Ginalski K, Rychlewski L, García J L, Díaz E (2008). Proc Natl Acad Sci U S A.

[64] Belda E, van Heck R G A, Lopez-Sanchez M J, Cruveiller S, Barbe V, Fraser C, Klenk H-P, Petersen J, Morgat A, Nikel P I (2016). Environ Microbiol.

[65] Wang H, Zhi X-Y, Qiu J, Shi L, Lu Z (2017). Front Microbiol.

[66] Wang S, Huang H, Xie K, Xu P (2012). Appl Microbiol Biotechnol.

[67] Ma Y, Wei Y, Qiu J, Wen R, Hong J, Liu W (2014). Appl Microbiol Biotechnol.

[68] Yu H, Tang H, Zhu X, Li Y, Xu P (2015). Appl Environ Microbiol.

[69] Huang H, Yu W, Wang R, Li H, Xie H, Wang S (2017). Sci Rep.

[70] Li J, Qian S, Xiong L, Zhu C, Shu M, Wang J, Jiao Y, He H, Zhang F, Linhardt R J (2017). Front Microbiol.

[71] Li H, Xie K, Yu W, Hu L, Huang H, Xie H, Wang S (2016). Appl Environ Microbiol.

[72] Fitzpatrick P F, Miller S, Hille R, Palfey B A (2013). Amine and amino acid oxidases and dehydrogenases. Handbook of Flavoproteins.

[73] Yu H, Li Y, Tang H, Xu P (2014). Genome Announce.

[74] Qiu J, Li N, Lu Z, Yang Y, Ma Y, Niu L, He J, Liu W (2016). Appl Microbiol Biotechnol.

[75] Qiu J, Wei Y, Ma Y, Wen R, Wen Y, Liu W (2014). Appl Environ Microbiol.

[76] Qiu J, Yang Y, Zhang J, Wang H, Ma Y, He J, Lu Z (2016). Front Microbiol.

